# Pelvic vein embolization: an assessment of the readability and quality of online information for patients

**DOI:** 10.1186/s42155-020-00143-0

**Published:** 2020-10-18

**Authors:** R. J. Lee, D. C. O’Neill, M. Brassil, J. Alderson, M. J. Lee

**Affiliations:** 1 Department of Radiology, Beaumont Hospital, Beaumont Rd, Dublin 9, Ireland; 2 RCSI, 123 St Stephens Green, Dublin, Ireland

**Keywords:** Pelvic vein, Embolization, Pelvic congestion syndrome, Information, Patient, Internet, Online

## Abstract

**Introduction:**

Pelvic congestion syndrome is a controversial topic. Pelvic vein embolization is a minimally invasive treatment for pelvic congestion syndrome. We aimed to assess the quality of information available on the Internet and determine how accessible information provided by the main IR societies was to patients.

**Materials and methods:**

The most commonly used term relating to pelvic vein embolization was searched across the five most-used English language search engines, with the first 25 web pages returned by each engine included for analysis. Duplicate web pages, nontext content and web pages behind paywalls were excluded. Web pages were analyzed for quality and readability using validated tools: DISCERN score, JAMA Benchmark Criteria, HONcode Certification, Flesch Reading Ease Score, Flesch–Kincaid Grade Level, and Gunning–Fog Index.

**Results:**

The most common applicable term was “Pelvic Vein Embolization”. Mean DISCERN quality of information provided by websites is “fair”. Flesh–Kincaid readability tests and Gunning–Fog Index demonstrated an average “college level” of reading ease. HON code certification was demonstrated in less than one third of web pages. Professional societies and scientific journals demonstrated the highest average JAMA and DISCERN scores, while for-profit organizations and healthcare providers demonstrated the lowest. Only information from 1 of 3 interventional societies was included in the first 25 search engine pages.

**Conclusion:**

The quality of information available online to patients is “fair” and outside of scientific journals the majority of web pages do not meet the JAMA benchmark criteria. These findings call for the production of high-quality and comprehensible content regarding interventional radiology, where physicians can reliably direct their patients for information.

## Introduction

In an ever-expanding technological age, there is a tendency for people to turn to the internet for information and advice on facets of everyday life including healthcare information (Marton and Wei 2012). Poor quality information can negatively influence patient decision making outside of the doctor-patient consultation as there is a broad range of health information ranging from patient experience discussed in online forums to more esoteric scientific journals (Papen 2013).

Interventional Radiology (IR) has revolutionized treatment for a wide range of conditions, including pelvic congestion syndrome (PCS) by offering pelvic vein embolization as a treatment. While pelvic vein embolization can offer a high success rate of symptom relief (Brown et al. 2018) it should be noted that treatment of PCS is a controversial area.

The primary purpose of this study was to assess the readability and quality of online information for patients with regard to pelvic vein embolization using a variety of online instruments and quality measures. Pelvic congestion syndrome was chosen because it is controversial and quality information would help patients navigate the problem and inform themselves. A secondary aim was to assess the information provided by large English-speaking IR societies such as the Cardiovascular and Interventional Society of Europe (CIRSE), the Society of Interventional Radiology (SIR) and the British Society of Interventional Radiology (BSIR).

## Materials and methods

### Web page selection process

Our study’s search strategy was similar to that instituted by Murray et al. (2018). A list of the most familiar search terms describing pelvic vein embolization was selected from both relevant literature and patient-information websites; Pelvic Vein Embolization, Ovarian Vein Embolization. Each of these terms was then searched across the five most popular English-language based search engines (Google, Bing!, Ask.com, Yahoo, and AOL Search) (Chris 2019). All searches were conducted from the same Internet Protocol address in a cache and cookie cleared manner to minimize the influence of previous queries. Only the top 25 web pages for each search engine were examined, as it has been illustrated that patients are unlikely to view beyond these results for health-related searches (O’Neill et al. 2014; Silberg et al. 1997). The exclusion criteria included web pages advertised by search engines, web pages with paywall access, sole video and audio content, geographically inaccessible web pages, duplicate web pages, or web pages that were subsections of others.

### Quality

Each web page was assessed for quality using three validated methods: the JAMA Benchmark Criteria, Health on the Net Foundation (HONcode) certification and the DISCERN instrument (O’Neill et al. 2014). The JAMA benchmark criteria are four criteria; (i) authorship, (ii) attribution of sources of information, (iii) disclosure of conflict of interest and (iv) how current the information is (Silberg et al. 1997). Publishing organization was recorded either from the web page itself or from the “About Us/Contact Us” section. The date of creation, or last reported update was recorded to assess for currency similar to a strategy used by Alderson et al. (2019).

The DISCERN instrument is a 16-point questionnaire that assesses important aspects of information reliability, description of treatment choices, and overall information quality (Charnock et al. 1999). A higher score indicates higher quality healthcare information. DISCERN has demonstrated inter-observer reliability and construct validity when used either by medical professionals or laypersons (Alderson et al. 2019; Rees et al. 2002).

HONCODE Health on the Net code of conduct (HONcode) is a website standard that assess the credibility and reliability of healthcare information. Prior studies have shown that HONcode is a marker of reliable medical information (Laversin et al. 2011) and is allied with superior clinical precision (Fallis and Fricke 2002). HONcode certification was recorded by checking the HONcode online database.

### Readability

The National Institute of Health and the American Medical Association recommend that the readability level of health information for patients should not surpass a 6th grade reading level (Weiss 2003). Readability was assessed using a number of assessment tools via an online analysis tool: (i) the Flesch Reading Ease Score (FRES), (ii) the Flesch-Kincaid Grade Level (FKGL), and (iii) the Gunning-Fog Index (GFI) (Readability Test Tool 2019). The FRES score reports the readability ease of a text on a scale from 0 to 100, with 100 being the easiest text to read and 0 being the most difficult. The FKGL formula is dependent on two variables: average sentence length and average number of syllables per word (Flesch 1948). The higher the score, the easier the passage is to read. These two scores correlate with a required level of education to read an item (US school grade) (Kincaid et al. 1975). The GFI is a separate readability measure that additionally accounts for word complexity and word unfamiliarity using the formula 0.4 [(words/sentences) + 100 (complex words/words)], with reference to a list of common words that are not considered complex, regardless of syllable count. This then estimates the number of years of education required to read an article (O’Neill et al. 2014; Walsh and Volsko 2008). The higher the GFI score, the more intricate the passage is to read. Additionally, qualification of author and web page owner were also collected.

### Statistical methods

Spearman rank order was used to assess the correlation between web page quality scores with their respective position in the order of search results. JAMA benchmark criteria score, mean web page age and DISCERN score were compared across different web page owners by one-way analysis of variance (ANOVA). Significance was predetermined at *p*-value < 0.05. Analysis was performed using Stata/IC 15 software (StataCorp 2017). An online statistics tool was used to create box plots.

Institutional Review Board (IRB) unnecessary as no human subjects involved in this study.

## Results

### Search terms

Analysis identified that Pelvic Vein Embolization was the most common search term (824,000 hits). The first 25 search results were chosen for analysis from each of the 5 search engines yielding a total of 125 items. Seventy-five search results were excluded from analysis: 55 duplicate web pages and 20 non-readable links (video *n* = 13, paywall access web pages *n* = 6 and website not accessible *n* = 1). Fifty search results remained for analysis (Table 1).

**Table 1:** Web page ownership by organisation type

Owner	Total (*n* = 50)
Healthcare Provider	22
Scientific Journal	11
Not-for-profit organisation	8
For-profit organisation	6
Professional Society	3

### JAMA benchmark criteria

Compliance with JAMA benchmarks was separately recorded for each website (Table 2). Only 14% of websites (*n* = 7) fulfilled a full JAMA Benchmark score of 4, all being from scientific journals.

**Table 2:** Compliance with the Journal of the American Medical Association (JAMA) Quality Benchmarks

JAMA Benchmark Criteria	Number	%
Authorship	26	52
Attribution	21	42
Currency	25	50
Disclosure	10	20

### Authors

Doctor(s) (*n* = 23) and non-medical authors (*n* = 3). In the remaining cases (*n* = 24), the author was not reported. 50% of websites provided the original date of publication or update (the most recent being recorded), with a mean age of 3.28 years.

### Currency

Scientific journals had 100% compliance (*n* = 11) whereas both for profit (*n* = 6) and non-profit (*n* = 8) organisations had 50%, professional societies 30% (*n* = 1) and lastly healthcare providers having a low compliance level of 27% (*n* = 6).

### DISCERN Score

Scientific Journals had the highest average DISCERN score of 51.2 while for profit organisations had the lowest score of 35 (Table 3). 48% of websites were rated between ‘very poor’ and ‘poor’ with a DISCERN score of less than 38, while 32% of articles achieved a score of ‘fair’ and only 20% receiving a ‘good’ score of 51–62. No websites scored well enough to be deemed excellent by the DISCERN tool rating (*p*-value < 0.0002) (Table 3).

**Table 3:** Discern Score-Quality of Websites

DISCERN Rating	Percentage (%)	Number of websites
Very Poor (16–26)	4	2
Poor (27–38)	44	22
Fair (39–50)	32	16
Good (51–62)	20	10
Excellent (> 63)	0	0
Total	100	50

### HONcode Certification

In total 10% of all websites had HONcode certification with scientific journals having the highest score of 6%, for-profit, non-profit and professional societies all having 2% certification and health care providers having no Honcode certification (Table 4).

**Table 4:** Summary of Results

Producer	Discern Score	JAMA Score	HONcode Certification (%)	FRES	FKGL	GFI
For Profit Organisation	35	0.83	2	40.3	12.2	15.3
Healthcare Provider	37.4	0.63	0	41.3	12.4	14.6
Professional Society	45.6	2.33	2	54	21.4	24.9
Non-Profit Organisation	38.4	1.87	2	47.9	11.5	14.4
Scientific Journal	51.2	3.63	6	33.5	14	16.1

All results are mean value

*JAMA* Journal of American Medical Association, *HONcode* Health on the Net Code, *FRES* Flesch Reading Ease Score, *FKGL* Flesch-Kincaid Grade level, *GFI* Funning-Fog Index

### Readability

In general, the average readability scores inclusive of the FKGL (13.1) (*p*-value 0.0412), GFI (15.6) (*p*-value 0.0322) and FRES (41.3) (*p*-value 0.0314), show values indicating a “college level” of reading ease. Table 4 demonstrates the variation by web-page owner.

### Quality assessment

Mean JAMA score was 3.28. JAMA scores varied significantly by web page owner (rs = − 0.2517, p (2-tailed) = 0.07786). Mean DISCERN score was 40.8. DISCERN scores did not vary significantly by web page owner (rs = − 0.1549, p (2-tailed) = 0.27777). Correlation between search ranking and quality score failed to reach significance, using both DISCERN score and JAMA bench- mark criteria.

### Readability assessment

The average FRES score was 41.30. The average FKGL score was 13.14. The average GFI score was 15.61. ANOVA between groups showed no significance between FRES FKGL, GFI score and web page owner (rs = 0.12, p (2-tailed) = 0.41 rs = − 0.18, p (2-tailed) = 0.21 and rs = − 0.14, p (2-tailed) = 0.33 respectively).

From patient information web pages provided by IR societies, only BSIR placed in the top 25 search results. CIRSE or SIR material was not identified in the top 25 search results in any of the search engines. The BSIR page showed a JAMA score of 2, a good Discern score of 54 and readability scores of 53.8 (FRES), 10.3 (FKGL) and 13.6 (GFI).

### The publishing journals of these societies showed

CVIR had 2 papers (4 search engine results with 2 being duplicates and one being paywall access) in the top 10 search results across all search engines. JVIR had the most results of all IR journals with 5 papers across Google and ask.com search engines. Two of these papers were paywall access.

## Discussion

Undoubtedly, Pelvic Congestion syndrome is a controversial topic in medicine. Within IR, there is no consensus on treatment approach as most of the literature is a collection of techniques and targetable therapeutic sites. Procedures are often not covered by insurance companies due to a lack of high quality literature. It is clear from review of these websites that there is a mix of information for patients to decipher. While some websites do note other treatment options for PCS including analgesia and surgical options, for profit standalone clinics and private surgical centers often only describe Pelvic Vein Embolization as the only effective curative approach with high success rates. The technique of embolization was also variably covered.

There was a diverse difference in web page quality as interpreted by JAMA and DISCERN scores. Professional Societies and Scientific journals showed the highest average scores. Of note scientific journals also scored the lowest FRES result, which signifies a more difficult text for the public to read. The more numerous, understandable web pages in the healthcare provider category, could easily tempt the general public to engage with the lower-quality information of these website providers.

Pages assessing JAMA benchmarks showed that most lacked authorship, references and disclosure. Doctors represented the largest group of authors (46%), however a further 48% of web pages did not report authors which questions the legitimacy of those web pages. The average DISCERN score was 40.8, meaning a “fair” quality which is below what many patients would expect to find when looking for information. As expected, scientific journals showed the highest average DISCERN score, and were the only category to fulfill all JAMA benchmark criteria. However, the target audience for scientific journals is not patients. The medical terminology and “jargon” used may actually make the information inaccessible to patients. Healthcare providers and for-profit organizations had the lowest JAMA and DISCERN scores, which suggests the provision of limited amounts of information to emphasize their own particular interests. An area all web pages could improve upon is HONcode certification.

In general, the average readability indicating a “college level” of reading ease. Thus, web pages were of both moderate difficulty in terms of word complexity and technical readability. To put that into perspective, this paper scores a 14.1 FKGL score, GFI score of 16.3 and FRES score of 32.8 which is representative of the difficult average readability of the information patients are accessing online when they search for pelvic vein embolization. As the general public increasingly rely on the Internet for access to information regarding procedures, it is important that the information available is at a suitable level. Instead of the suggested 6th grade readability level for health information (Weiss 2003), we have shown the level to be significantly higher.

Web-based health information is critical and can alter behavior, reach peers in real time, increase satisfaction with care, improve health outcomes, and facilitate shared decision-making between patients and healthcare professionals of the ever-expanding proportion of the population relying on the Internet for information (Suggs 2006; Daraz et al. 2011).

Better understanding of IR procedures would likely encourage and foster a more appropriate and well-informed decision-making process. Effective communication and propagation of quality information relating to interventional procedures is important for the continued expansion of Interventional Radiology as a specialty, as well informed patients are more likely to choose less invasive treatment options (Becker 2001). Many independent bodies and IR societies (CIRSE, SIR, BSIR) have patient information web pages, however these pages may not be found by patients due to their inclination not to proceed past the first 25 results. In fact, both CIRSE and SIR information pages on pelvic vein embolization do not make it into the top 100 Google search results. These organizations should aim to increase their online presence by moving higher up on the search engine ranking.

### Study limitations

Readability tools often do not take into account the content or complexity of medical vocabulary or patients’ familiarity with medical terminology and may underestimate or overestimate the actual readability of online health information (Smith et al. 2011; Pichert and Elam 1985). Scoring was performed by doctors rather than patients who embody the target demographic in this study. This paper did not assess multimedia websites.

### Future work

One intriguing area for future research relates to the use of mixed online media (e.g., video, audio) to deliver health care information. Mixed multimedia health information may be easier to understand than traditional text.

## Conclusion

The reliability and quality of online content remains a critical issue for patients and doctors alike. This study demonstrates that outside of scientific journals, the majority of web pages do not meet the JAMA benchmark criteria. Overall, most patients would find it difficult to understand these articles, have little measure of which articles to trust and could be misled by the quality of content within. Content producers on the Internet need to have increased awareness of quality and readability tools which, when applied, could improve their trustworthiness and patient’s understanding. We believe that there is a necessity for a high-quality Interventional Radiology website, that is current, impartial, easy to read and well sourced at an accessible level for patients.

## Data Availability

The datasets used and/or analysed during the current study are available from the corresponding author on reasonable request.
